# Comprehensive Evaluation of Non-invasive Prenatal Screening to Detect Fetal Copy Number Variations

**DOI:** 10.3389/fgene.2021.665589

**Published:** 2021-07-16

**Authors:** Jing Wang, Bin Zhang, Lingna Zhou, Qin Zhou, Yingping Chen, Bin Yu

**Affiliations:** Changzhou Maternity and Child Health Care Hospital Affiliated to Nanjing Medical University, Changzhou, China

**Keywords:** non-invasive prenatal screening, copy number variant, chromosome microarray analysis, prenatal screening, positive predict value, detection rate

## Abstract

**Objective:**

To evaluate the effectiveness of non-invasive prenatal screening (NIPS) in prenatal screening of fetal pathogenic copy number variants (CNVs).

**Materials and Methods:**

We evaluated the prenatal screening capacity using traditional and retrospective approaches. For the traditional method, we evaluated 24,613 pregnant women who underwent NIPS; cases which fetal CNVs were suggested underwent prenatal diagnosis with chromosomal microarray analysis (CMA). For the retrospective method, we retrospectively evaluated 47 cases with fetal pathogenic CNVs by NIPS. A systematic literature search was performed to compare the evaluation efficiency.

**Results:**

Among the 24,613 pregnant women who received NIPS, 124 (0.50%) were suspected to have fetal CNVs. Of these, 66 women underwent prenatal diagnosis with CMA and 13 had true-positive results. The positive predictive value (PPV) of NIPS for fetal CNVs was 19.7%. Among 1,161 women who did not receive NIPS and underwent prenatal diagnosis by CMA, 47 were confirmed to have fetal pathogenic CNVs. Retesting with NIPS indicated that 24 of these 47 cases could also be detected by NIPS, representing a detection rate (DR) of 51.1%. In total, 10 publications, namely, six retrospective studies and four prospective studies, met our criteria and were selected for a detailed full-text review. The reported DRs were 61.10–97.70% and the PPVs were 36.11–80.56%. The sizes of CNVs were closely related to the accuracy of NIPS detection. The DR was 41.9% (13/31) in fetuses with CNVs ≤ 3 Mb, but was 55.0% (11/20) in fetuses with CNVs > 3 Mb. Finally, to intuitively show the CNVs accurately detected by NIPS, we mapped all CNVs to chromosomes according to their location, size, and characteristics. NIPS detected fetal CNVs in 2q13 and 4q35.

**Conclusion:**

The DR and PPV of NIPS for fetal CNVs were approximately 51.1% and 19.7%, respectively. Follow-up molecular prenatal diagnosis is recommended in cases where NIPS suggests fetal CNVs.

## Introduction

Redon et al. (2006) constructed the first-generation copy number variant (CNV) map and reported that CNVs were ubiquitous in the human genome. Some CNVs exist in the normal human population in the form of genetic polymorphisms, while others may be related to human traits and diseases. With developments in sequencing technology, studies have increasingly shown that CNVs can lead to genetic diseases and syndromes due to gene dosage effects, gene fracture, gene fusion, and location effects (e.g., Mendelian single-gene diseases, rare diseases, and complex diseases). Studies of prenatal diagnosis have shown that pathogenic CNVs are associated with adverse pregnancy outcomes (Watson et al., 2014; Srebniak et al., 2016). The generation of CNVs is very common and can occur randomly at any location on the chromosome. Fetal genetic diagnosis continues to rely on invasive procedures, and no effective non-invasive prenatal screening (NIPS) methods are currently available.

Non-invasive prenatal screening is widely used to screen for fetal chromosome trisomies 21, 18, and 13 (T21, T18, and T13, respectively), as well as sex chromosome aneuploidy (SCA). Many clinical applications have confirmed the accuracy of this method (Norton and Wapner, 2015; Gil et al., 2017). The detection rate (DR), specificity, and false-positive rate (FPR) of NIPS for T21, T18, and T13 were reportedly 98.59, 99.99, and 0.02%, respectively (Yu et al., 2017). The overall positive predictive values (PPV) were reportedly 65–94% for T21, 47–85% for T18, 12–62% for T13, and 45–58% for SCA (Quezada et al., 2015; Neofytou et al., 2017; Zhang et al., 2017; Zheng et al., 2020; Wang et al., 2020). Recently, NIPS was described as a contributing factor in the detection of fetal genomic CNVs (Li et al., 2016) and monogenic inherited diseases (Xu et al., 2015). With the development of high-throughput sequencing technology, NIPS has attracted attention for fetal CNV prenatal screening. However, previous studies focused on limited types of multiple marker screening (Liang et al., 2019). Most recent reports have evaluated its PPV alone because of the difficulty in evaluation of DR. It is challenging to identify microdeletions and microduplications in neonates by routine follow-up after prenatal screening due to delayed clinical manifestations. Despite the appearance of clinical symptoms, it may not be possible to obtain a timely diagnosis.

In this study, we evaluated the PPV using the traditional process (NIPS screening, then prenatal diagnosis), then conducted a retrospective study to evaluate the DR and false-negative rate (FNR) of NIPS for detection of fetal CNVs. Factors that may affect NIPS detection efficiency were identified based on analysis of the results.

## Materials and Methods

### Ethics Approval and Consent to Participate

The study design and protocol were reviewed and approved by the ethics committee of Changzhou Maternity and Child Health Care Hospital affiliated to Nanjing Medical University (No. 2017003). All pregnant women received genetic counseling and provided informed consent before testing.

### Clinical Subjects

#### Traditional Process

**FIGURE 1:**
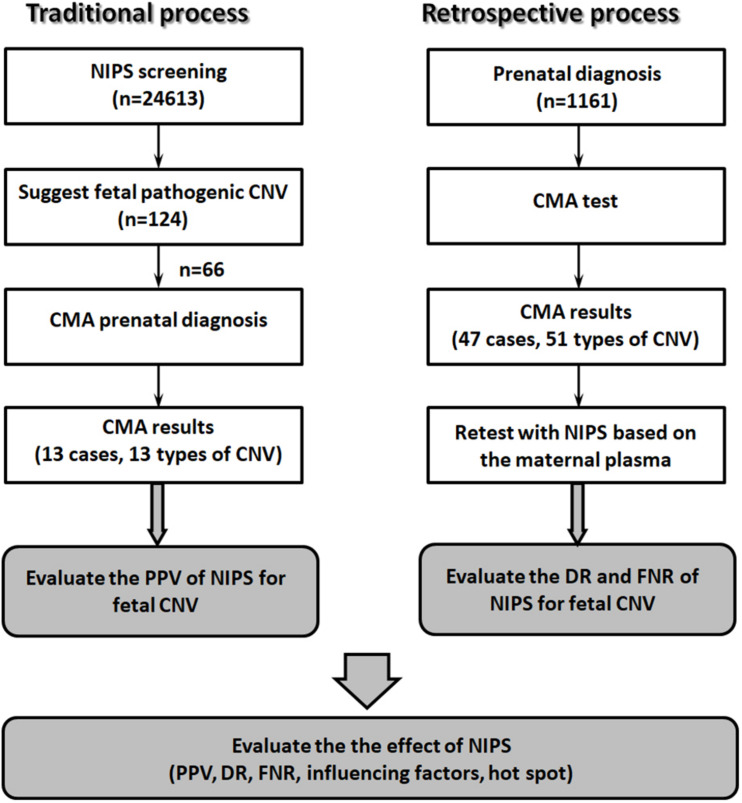
Schematic of the study design.

From January 2019 to July 2020, 24,613 pregnant women underwent NIPS at our hospital. In total, 124 cases were suspected to have fetal CNVs. The pregnant women had singleton pregnancies, were 19–45 years old (mean age, 31.2 years), and were at gestational weeks 14^+1^–25^+2^ (mean gestational age, 17.1 weeks). After genetic counseling, 66 women received confirmatory invasive prenatal diagnosis with chromosomal microarray analysis (CMA) after amniocentesis (Figure 1).

#### Retrospective Process

A total of 1,161 pregnant women with abnormal fetal ultrasound findings, and at high risk, as determined by prenatal serological screening, advanced maternal age, and adverse reproductive outcomes (other than abnormalities suspected by NIPT) underwent prenatal diagnosis by CMA; fetal pathogenic CNVs were detected in 47 cases. All included women were 19–49 years old (mean, 31.4 years). The range of gestational age was 17–33 weeks (mean, 23.2 weeks). Maternal blood samples were collected before amniocentesis. The plasma was isolated from the blood samples, stored at −80°C, and retrospectively analyzed for NIPS (Figure 1).

### NIPS

Peripheral blood (5 ml) was collected by simple needle aspiration in all cases and plasma was isolated within 48 h of collection. Plasma DNA was extracted using a QIAamp Circulating Nucleic Acid Kit (Qiagen, Hilden, Germany), in accordance with the manufacturer’s instructions. Sequencing library construction, quality control, and multiplexing for sequencing were performed as described previously (Shi et al., 2019). DNA libraries were constructed from 1.2 ml of purified DNA from maternal plasma following the manufacturer’s protocol (Berry Genomics Co., Ltd.) and subjected to massively parallel sequencing (NextSeq CN500 platform; Illumina, San Diego, CA, United States), One lane of the Illumina Next CN500 v2 flow cell was used to perform sequencing with a single-ended 43-bp sequencing protocol, in accordance with the manufacturer’s instructions. Based on the NIPS detection platform, we recorded fetal microdeletions/microduplications for > 1 Mb CNVs.

### Prenatal Diagnosis by CMA

The procedure of prenatal CMA was described in our previous study (Shi et al., 2019). Amniotic fluid was collected and DNA was extracted using a QIAamp DNA Mini Kit (Qiagen Inc., Valencia, CA, United States). Aliquots of 250 ng of DNA were amplified, labeled, and hybridized using the GCS 3000Dx v.2 platform (Affymetrix, Santa Clara, CA, United States). SNP array tests were performed using a commercial 750 K microarray chip (CytoScan 750 K Array; Affymetrix). After hybridization with fragmented DNA, the chip was washed with buffer and scanned by an Alaser scanner. The data were analyzed using the Chromosome Analysis Suite v3.2 (ChAs) software package (Affymetrix). Public databases (e.g., DECIPHER, OMIM, ClinVar, ISCA, NCBI, and UCSC) were used to interpret the data. The pathogenicities of identified CNVs were evaluated in accordance with the guidelines of the American College of Medical Genetics and Genomics.

### Clinical Consultation and Follow-Up

All pregnant women included in the study received prenatal genetic counseling. The women undergoing NIPS were recalled by a trained specialist if the screening results indicated fetal CNVs. After the reception of prenatal genetic counseling from a professional geneticist, the women underwent prenatal diagnosis by CMA after amniocentesis at gestational weeks 18–26. Additionally, the pregnancy outcomes of all women were followed up by telephone and local health information system tracking.

### Systematic Literature Search

A systematic literature search was performed to compare the efficiency of NIPS for detecting fetal CNVs among current research methods. We searched PubMed^1^ for all published cohort studies regarding NIPS detection of fetal CNVs from January 2016 to December 2020 using the following search string: (“NIPS” OR “non-invasive prenatal testing” OR “fetal copy number variations” “whole-genome sequencing” OR “chromosomal abnormalities” OR “microduplication” OR “microdeletion”) NOT review [pt] AND English [la].

### Statistical Analysis

The data were analyzed using Empower Stats software (X&Y Solutions, Inc.) and R^2^ (Yu et al., 2018). The chi-squared test was used to compare differences in continuous variables between two groups. In all analyses, *p* < 0.05 was considered to indicate statistical significance.

## Results

### Evaluation of NIPS for Detecting Fetal CNVs

We evaluated the PPV of NIPS for fetal CNVs using the traditional process. Among the 24,613 prenatal women receiving NIPS, 124 (0.50%) were suspected to have fetal CNVs ranging in size from 1.3 Mb to 82 Mb, namely, 28 cases of microdeletions and 96 cases of microduplications. After genetic counseling, 66 women underwent prenatal diagnosis with CMA test (rate of prenatal diagnosis, 53.2%). As shown in Table 1, 13 of the 66 cases had true-positive results. Thus, the PPV of NIPS for fetal CNVs was 19.7% (31.6% for microdeletions and 14.9% for microduplications). There were no significant differences between the two groups (*p* > 0.05).

**TABLE 1:** Results of CMA prenatal diagnosis after NIPS.

Group	*n*	Prenatal diagnosis rate (%)	CMA			PPV (95% CI)
			*n*	TP	FP	
NIPS suspected microdeletion	28	67.9	19	6	13	31.6% (13.6–56.6%)
NIPS suspected microduplication	96	49.0	47	7	40	14.9% (6.7–28.9%)
Total	124	53.2	66	13	53	19.7% (11.3–31.7%)

In the retrospective process, we evaluated the DR and FNR. The 1,161 women who did not receive NIPS underwent prenatal diagnosis by CMA; 47 were confirmed to have fetal pathogenic CNVs, namely, 22 cases with fetal microdeletions, 22 cases with microduplications, and 3 cases with both microdeletions and microduplications (Table 2). Maternal plasma was collected before amniocentesis and retested with NIPS. The results showed that 24 of these 47 cases could also be detected by NIPS, representing a DR of fetal CNV by NIPS of 51.1% (Table 2). The DRs were 40.9% (9/22), 59.1% (13/22), and 66.7% (2/3) for fetal microduplications, microdeletions, and multiple-segment microdeletions/microduplications, respectively. There were no significant differences among groups (*p* > 0.05).

**TABLE 2:** Results of NIPS in CMA prenatal diagnosis.

Group	*n*	NIPS		DR (95% CI)	FNR (95% CI)
		Detected	Undetected		
CMA indicated microdeletion	22*	13	9	59.1% (36.7–78.5%)	40.9% (21.5–63.3%)
CMA indicated microduplication	22	9	13	40.9% (21.5–63.3%)	59.1% (36.7–78.5%)
CMA indicated microdeletion and microduplication	3	2	1	66.7% (12.5–98.2%)	33.3% (1.8–87.5%)
Total	47	24	23	51.1% (36.3–65.7%)	48.9% (34.3–63.8%)

*One case indicated as two microdeletion CNVs.

### Systematic Literature Search

In total, 10 papers (six retrospective studies and four prospective studies) met our criteria and were selected for detailed full-text review (Lefkowitz et al., 2016; Li et al., 2016; Liu et al., 2016; Cui et al., 2019; Hu et al., 2019; Liang et al., 2019; Yu et al., 2019; Hyblova et al., 2020; Ye et al., 2020; Yin et al., 2020). We summarized and analyzed the research methods, sample size, sequencing depth, platform, algorithm, DR, specificity, and PPV. Most performed retrospective NIPS testing on pregnant women with fetal CNV cases confirmed by prenatal diagnosis, mainly to evaluate the DR and specificity (Table 3). The study sample sizes ranged from 34 to 1,222 cases. The reported DRs were 61.10–97.70%, based on different algorithms and sequencing depths. The specificity was > 95% in all cases. A single paper reported the PPV (90.91%), but no information was provided regarding its calculation (Cui et al., 2019). The prospective studies identified in the search had larger sample sizes, ranging from 6,239 to 94,085 cases. The PPV ranged from 36.11% to 80.56%. All articles also reported the DR, which ranged from 36.11 to 90.74%. However, with such large numbers of clinical cases, it is difficult to confirm the cases of fetal CNVs in a timely manner, and it is insufficient to rely on routine follow-up methods. Therefore, we could not determine the sources of these DR calculations. Here, we combined the two study strategies and achieved DR of 51.1% and PPV of 19.7%. These results were lower than in the literature, which may have been related to differences in algorithms, methods, and platforms used between studies.

**TABLE 3:** Relevant literature.

Study	Depth	Sample number	NIPS positive	CNVs confirmed	DR	Specificity	PPV	Platform	Method/Algorithm	Study design
Liu et al., 2016	Low	919	–	33	84.21	98.42	–	Illumina HiSeq2000	FCAPS	Retrospective
Li et al., 2016	Low	117	–	18	61.1	95	–	Illumina HiSeq	MPS	Retrospective
Lefkowitz et al., 2016	Increased	1,222	43*	35	97.7	99.9	–	–	CBS	Retrospective
Cui et al., 2019	Low	161	9	10	83.33	99.34	90.91	Illumina NextSeq CN500	–	Retrospective
Hyblova et al., 2020	Low	34	32	29	90.62	–	–	Illumina NextSeq 500/550	CBS	Retrospective
Ye et al., 2020	Low	873	32	48	67.31	97.45	–	Burrows-Wheeler aligner (BWA)	FCAPS	Retrospective
Yu et al., 2019	Low	20,003	36	29	80.56	–	80.56	Illumina NextSeq 550	CBS	Prospective
Hu et al., 2019	Low	8,141	51	13	36.11	–	36.11	JingXin BioelectronSeq 4000	MPS	Prospective
Liang et al., 2019	Increased	94,085	163	120	90.74	99.924	40.8	Illumina NextSeq CN550	RUPA	Prospective
Yin et al., 2020	Low	6,239	48	32	67.7	–	–	Ion Proton Sequencing System	–	Prospective
This study	Low	24,613	66	13	–	–	19.7	Illumina Nextseq CN500	RUPA	Prospective
	Low	1,161	24	47	51.1	–	–			Retrospective

*Includes both sub-chromosomal CNVs (Lo et al., 2016) and whole chromosome trisomies other than T13, T18, T21, and SCAs. FCAPS, fetal copy-number analysis through maternal plasma sequencing; CBS, circular binary segmentation; MPS, massively parallel sequencing.

### Factors Influencing CNV Detection Efficiency by NIPS

As shown in Table 4, the size of CNVs seemed to be closely related to the accuracy of NIPS detection. In the retrospective process, 51 types of fetal pathogenic CNVs ranging in size from 0.5 to 26.5 Mb were detected by CMA in 47 cases. We analyzed the fragments according to their size. The DR was 41.9% (13/31) in fetuses with CNVs ≤ 3 Mb, but was 55.0% (11/20) in fetuses with CNVs > 3 Mb. The DR was highest (85.7%) in fetuses with CNVs ≥ 10 Mb. There were significant differences between groups (*p* = 0.03 and *p* < 0.05). The DR of NIPS for CNVs ≥ 10 Mb in this study was similar to the DR reported previously (Akolekar et al., 2015). A single case with a CNV size of 11 Mb was not detected, which was located at 11q24.2q25, fetal fraction was 12.1%, and UniMaprds was 2.5 M. However, the detection ability of NIPS did not seem to be closely related to fetal fraction or UniMaprds. In the present study, the fetal fraction ranged from 5.5% to 38.5%, and the UniMaprds ranged from 2.2 to 4.8 Mb. The DRs were 52.9% and 46.2% in the groups with fetal fraction ≥ 10% and < 10%, respectively (*p* > 0.05), and 54.2% and 47.8% in the groups with UniMaprds ≤ 3 Mb and > 3 Mb, respectively (*p* > 0.05).

**TABLE 4:** Factors influencing NIPS detection efficiency.

CMA	*n*	NIPS	
		DR (% [*n*/*N*])	FNR (% [*n*/*N*])
		(95% CI)	(95% CI)
**CNVs size**			
CNVs ≤ 3 Mb	31	41.9 (13/31)	58.1 (18/31)
		(25.1–60.7%)	(39.3–74.9%)
3 Mb < CNVs < 5 Mb	8	50.0 (4/8)	50.0 (4/8)
		(17.4–82.6%)	(17.4–82.6%)
5 Mb ≤ CNVs < 10 Mb	5	1/5	4/5
		(1.1–70.1%)	(29.8–98.9%)
CNVs ≥ 10 Mb	7	85.7 (6/7)	14.3 (1/7)
		(42.0–99.2%)	(0.8–58.0%)
**Fetal fraction (%)**			
4–10	13	46.2 (6/13)	53.8 (7/13)
		(20.4–73.9%)	(26.1–79.6%)
≥10	34	52.9 (18/34)	47.1 (16/34)
		(35.4–69.8%)	(30.2–64.6%)
**UniMaprds (M)**			
≤3 Mb	24	54.2 (13/24)	45.8 (13/24)
		(33.2–73.8%)	(26.2–66.8%)
>3 Mb	23	47.8 (11/23)	52.2 (12/23)
		(27.4–68.9%)	(31.1–72.6%)

### Locations of CNVs Detected by NIPS

**FIGURE 2:**
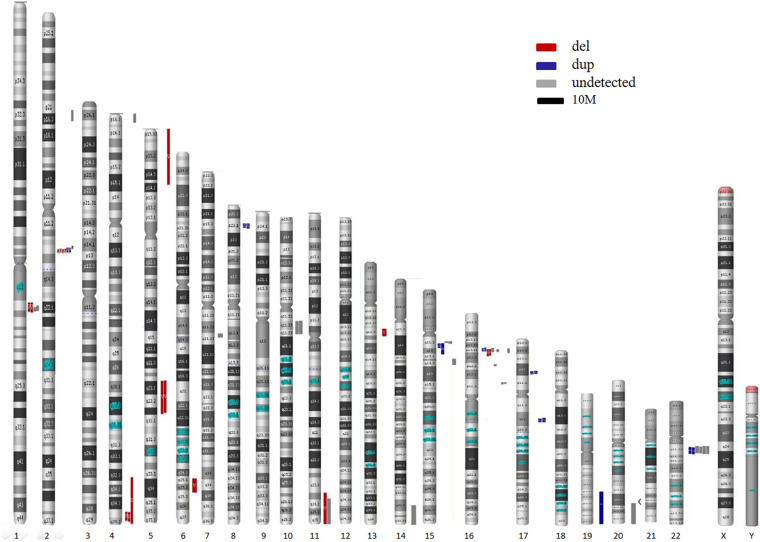
Map of all CNVs identified in the present study.

In the present study, 55 CNVs were detected by both NIPS and/or CMA, namely, 13 in traditional studies and 42 in retrospective studies (excluding 9 on the X chromosome) (Table 5). In total, 32 CNVs were successfully detected by NIPS, namely, 13 by the traditional process and 19 in retrospective studies. In addition, 23 types of CNVs were not detected by NIPS but were confirmed by prenatal CMA diagnosis. All CNVs were pathogenic and were located on chromosomes 1, 2, 4, 7, 10, 11, 14, 15, 16, 20, and 22. The fragment size ranged from 0.4 to 26.5 Mb. To intuitively show the CNVs accurately detected by NIPS, we mapped all CNVs to chromosomes according to their location, size, and characteristics. As shown in Figure 2, the fetal CNVs detected in this study involved 17 autosomes. The three most common loci for the incidences of fetal pathogenic CNVs were 16p11-13, 2q13, and 22q11, followed by 15q11-13, 1q21, and 4q35. All fetal CNVs in 2q13 and 4q35 could be detected by NIPS with fragment sizes between 1.7 and 2.0 Mb and between 4.5 and 22 Mb, respectively. NIPS also detected 4/10 fetuses with CNVs in 16p11-13, 2/4 in 1q21, and 2/5 in 15q11-13. The DR of CNVs in 22q11 was unsatisfactory; among six cases, we detected only two fetal CNVs by NIPS, both of which had a 2.8-Mb microduplication in 22q11.

**TABLE 5:** Detailed information regarding fetal CNVs in this study (detected or not detected by NIPS).

Chromosome	*n*	Size (Mb)	NIPS					
			Detected (*n*)	CNVs		Undetected (*n*)	CNVs	
				Location	Size (Mb)		Location	Size (Mb)
Chr1	4	1.3–4	2	del 1q21.1	4.0	2	dup 1q21.1q21.2	2.0
				del 1q21.1	4.1		dup 1q21	1.3
Chr2	7	1.7–4.5	6	del 2q13	1.7	1	del 2p21p16.3	4.5
				dup 2q13	2.0			
				del 2q13	1.7			
				dup 2q13	1.8			
				del 2q13	1.7			
				del 2q13	1.8			
Chr4	4	3.5–22	3	del 4q32.3q35.2	22.0	1	del 4p16.3	3.5
				del 4q35.1q35.2	4.5			
				del 4q35.1q35.2	4.7			
Chr5	3	14.7–26.5	3	del 5q23.1	14.7			
				del 5p15.33	26.5			
				del 5q23.1q23.3	15.6			
Chr6	1	6.4	1	del 6q25.1	6.4			
Chr7	1	1.6	0	0	0.0	1	dup 7q11.23	1.6
Chr8	2	2.0–2.2	2	dup 8p23.1	2.0			
				dup 8p23.1	2.2			
Chr10	2	5.5	0	0	0.0	2	loss 10q11.22	5.5
Chr11	2	11	1	del 11q24.1q25	13.2	1	del 11q24.2q25	11.0
Chr13	1	3.3	1	loss 13q12.3q13.1	3.3	0		
Chr14	1	8.3	0		0.0	1	loss 14q32.2q32.33	8.3
Chr15	5	0.4–4.9	2	dup 15q11q13	4.9	3	del 15q11.2	1.9
				dup 15q11.2	1.6		dup 15q13.3	2.5
							del 15q11.2	0.4
Chr16	10	0.7–2.8	4	del 16p13.11	2.8	6	dup 16p11.2	0.7
				dup 16p13.11	1.4		del 16p13.11	1.6
				dup 16p13.11	1.4		dup 16p11.2	0.6
				del 16p13.11	2.7		dup 16p13.11	0.8
							del 16p12.2	0.7
							dup 16p13.11	1.7
Chr17	4	1.5–1.9	4	dup 17q12	1.9			
				dup 17q12	1.5			
				dup 17p12	1.4			
				dup 17p12	1.4			
Chr19	1	14.7	1	dup 19q13.31	14.7			
Chr20	1		0			1	dup 20q13.2	9.0
Chr22	6	2.8–3.2	2	dup 22q11.21	2.8	4	dup 22q11.21	3.2
				dup 22q11.21	2.8		del 22q11.21	3.2
							dup 22q11.21	2.8
							dup 22q11.21	2.8
Total	55		32			23		

## Discussion

At present, the prenatal diagnosis of fetal CNVs continues to rely on invasive testing procedures, such as amniocentesis and chorionic villus sampling, followed by karyotyping or microarray analysis. Although the risks of these invasive procedures are small, they cannot be ignored (Akolekar et al., 2015). Refusal to undergo invasive prenatal diagnosis may result in the failure to identify fetuses with CNV abnormalities. This study provides a basis for prenatal diagnosis and consultation. In this study, fetal CNVs were analyzed using NIPS based on screening for common aneuploidy without increasing the sequencing depth. The effectiveness of NIPS in prenatal screening of fetal pathogenic CNVs was evaluated by two strategies: traditional and retrospective. Finally, approximately 0.50% of pregnant women who received NIPS were suspected to have abnormal fetal CNVs, with DR of 51.1% and PPV of 19.7%.

Our results showed that the PPV of NIPS for fetal pathogenic CNVs was 19.7%, which was lower than the PPV in other clinical studies that reported values ranging from 36.1 to 40.8% (Hu et al., 2019; Liang et al., 2019). This discrepancy may have been due to differences in algorithms/methods and platforms used between studies. Deeper sequencing may reduce the number of false-positives (Gross et al., 2015). The high FPR of fetal CNV detection will result in an increased frequency of unnecessary invasive procedures. Nevertheless, we support the application of NIPS for fetal CNVs. As the ACMG states, there are no other screening options available to identify fetal CNVs (Gregg et al., 2016). Furthermore, CNV abnormalities are observed in 1.0–1.7% of pregnancies, independently of maternal age (Callaway et al., 2013). They are usually associated with chromosomal diseases, most of which cannot be detected by routine ultrasound scans, including syndromes associated with mental disability, developmental delay, and autism spectrum disorders (Battaglia et al., 2013; Ferreira et al., 2016).

In the retrospective study, we analyzed the efficiency of NIPS for the detection of fetal CNVs. Among 47 samples positive on CMA analysis, 51.1% (24/47) of fetal CNVs could be detected by NIPS. The consistency rates of CNV location and size were 100% and 83.3%, respectively. The sensitivities differed markedly in other retrospective studies, ranging from 61.1 to 84.2% (Lefkowitz et al., 2016; Li et al., 2016; Liu et al., 2016; Cui et al., 2019; Hu et al., 2019; Hyblova et al., 2020; Ye et al., 2020). The DR differences may be attributed to the limited sample size in previous studies, as well as marked differences in CNV size and location. In addition, some studies used deeper sequencing methods or algorithms to improve the DR (Chen et al., 2013, 2015; Srinivasan et al., 2013; Chu et al., 2014; Ladislav et al., 2014; Lo et al., 2016). These data show that the NIPS can also detect fetal CNVs with good performance, with the exception of aneuploidy.

In addition, the results of the present study indicated that the detection efficiency of NIPS had minimal correlations with fetal fractions and UniMaprds, but was closely related to the size of CNVs with NIPS, thus indicating more accurate detection of larger CNV fragments, as in previous reports. Li et al. (2016) reported that NIPS showed greater performance when detecting large CNVs, while its ability to detect CNVs of smaller size was reduced. Recent studies have shown that NIPS has good detection performance for fetal CNVs, especially for CNVs > 10 Mb (Liu et al., 2016; Yu et al., 2019). When the sequencing depth is increased, NIPS can also achieve satisfactory performance in detecting smaller CNVs, and the range of detectable CNV size can reach 2–7 Mb (Lefkowitz et al., 2016; Ye et al., 2020). However, the results presented here showed some discrepancies with previous studies, suggesting that in addition to the ability to detect CNVs was strongly dependent on the size of the CNVs, some specific CNVs are associated with the fetal fraction (Benn, 2016). In our study, we could not analyze the correlation between the DR of a certain microdeletion/microduplication and the relevant fetal fraction due to the small sample size.

Here, we assumed that NIPS shows preferential CNV detection according to location. The three most common loci for the incidences of fetal pathogenic CNVs were 16p11-13, 2q13, and 22q11, followed by 15q11-13, 1q21, and 4q35. The detection efficiencies may be greater for regions 2q13 and 4q35. NIPS could detect most cases of fetal CNVs in these regions, including CNVs < 3 Mb. Surprisingly, the DR of CNVs in 22q11 was unsatisfactory, in contrast to the published findings; among six cases, we detected only two fetal CNVs by NIPS. Unfortunately, due to the small sample size, we were unable to evaluate the DR and PPV of each CNV segment. Our literature search did not reveal accurate CNV region data; thus, we were unable to perform a meta-analysis. Furthermore, because of the random nature of CNVs, it is difficult to predict the complete chromosomal location preference. Ideally, NIPS should detect CNVs throughout the genome, predict the DR in different regions, and improve the technique with a very low FPR. Larger validation studies are required to obtain more accurate information regarding location preference. In addition, variations among laboratories, platforms, algorithms, and reagents may reveal different preferences.

## Conclusion

The DR and PPV of NIPS for fetal CNVs were approximately 51.1% and 19.7%, respectively. The screening effectiveness was closely related to the size and region of CNV fragments; larger CNVs could be detected more accurately by NIPS. Although the DR of low-depth NIPS for fetal CNVs was unsatisfactory, based on the harmfulness and screening status of pathogenic CNVs, follow-up molecular prenatal diagnosis remains important.

## Data Availability Statement

The original contributions presented in the study are included in the article/supplementary material, further inquiries can be directed to the corresponding author.

## Ethics Statement

The study design and protocol were reviewed and approved by the Ethics Committee of Changzhou Maternity and Child Health Care Hospital affiliated to Nanjing Medical University (No. 2017003). The patients/participants provided their written informed consent to participate in this study.

## Author Contributions

BY and JW carried out the assays and participated in the study design. JW, BZ, LZ, QZ, and YC carried out clinical consultations and laboratory tests and performed the statistical analysis. BY and BZ conceived the study, participated in its design and coordination, and helped draft the manuscript. All authors contributed to the article and approved the submitted version.

## Conflict of Interest

The authors declare that the research was conducted in the absence of any commercial or financial relationships that could be construed as a potential conflict of interest.

## Footnotes

1 https://pubmed.ncbi.nlm.nih.gov/

2 http://www.R-project.org
